# Calculation of Self, Corrected, and Transport Diffusivities of Isopropyl Alcohol in UiO-66

**DOI:** 10.3390/nano13111793

**Published:** 2023-06-02

**Authors:** Chinmay V. Mhatre, Jacob J. Wardzala, Priyanka B. Shukla, Mayank Agrawal, J. Karl Johnson

**Affiliations:** 1 Department of Chemical and Petroleum Engineering, University of Pittsburgh, Pittsburgh, PA 15260, USA; 2 TCAD, TSMC Technoloy Inc., San Jose, CA 95051, USA

**Keywords:** molecular dynamics, diffusion coefficients, metal organic frameworks, hydrogen bonding

## Abstract

The UiO-6x family of metal-organic frameworks has been extensively studied for applications in chemical warfare agent (CWA) capture and destruction. An understanding of intrinsic transport phenomena, such as diffusion, is key to understanding experimental results and designing effective materials for CWA capture. However, the relatively large size of CWAs and their simulants makes diffusion in the small-pored pristine UiO-66 very slow and hence impractical to study directly with direct molecular simulations because of the time scales required. We used isopropanol (IPA) as a surrogate for CWAs to investigate the fundamental diffusion mechanisms of a polar molecule within pristine UiO-66. IPA can form hydrogen bonds with the $\mu_{3}$-OH groups bound to the metal oxide clusters in UiO-66, similar to some CWAs, and can be studied by direct molecular dynamics simulations. We report self, corrected, and transport diffusivities of IPA in pristine UiO-66 as a function of loading. Our calculations highlight the importance of the accurate modeling of the hydrogen bonding interactions on diffusivities, with about an order of magnitude decrease in diffusion coefficients when the hydrogen bonding between IPA and the $\mu_{3}$-OH groups is included. We found that a fraction of the IPA molecules have very low mobility during the course of a simulation, while a small fraction are highly mobile, exhibiting mean square displacements far greater than the ensemble average.

## 1. Introduction

Metal-organic frameworks (MOFs) are a group of nanoporous materials with an array of promising applications, including gas detection, separations, and catalysis [1,2,3,4]. MOFs are composed of metal secondary building units (SBUs) and organic linker building blocks, allowing them to be arranged in a highly modular fashion. One particular application is the capture and degradation of chemical warfare agents (CWAs), which is an active field of research. Multiple studies show that the Zr-based MOFs are effective in CWA degradation [5,6,7,8,9,10,11,12]. UiO-66, a MOF composed of a Zr${}_{6}$O${}_{4}$(OH)${}_{4}$ SBU connected by benzene dicarboxylate (BDC) linkers, is one of the most promising MOFs for this application. Reactions of CWAs and simulants with UiO-66 have been studied with both experiments [13,14,15,16,17] and computations [18]. An understanding of the diffusion and adsorption of guest molecules in UiO-66 is critical to learning how they can be best designed to capture CWAs. Compared to other MOFs, UiO-66 and its derivatives are promising for CWA capture and degradation. High porosity with excellent physical, chemical, and water stability and available Zr catalytic sites makes them promising candidates [6,14]. Usually, UiO-66 and other Zr-based MOFs have metal centers acting as a strong Lewis acid site for degradation. The catalytic action relies on missing linker defects within within UiO-66 in order to create open metal sites because it is fully coordinated in the pristine state. UiO-66 has been shown to be amphoteric in many situations, allowing it to serve both roles in an acid–base-catalyzed reaction [19]. Yet, exploring the pristine structure for diffusion properties can still yield important insights. As UiO-66 has small diameter pores, ranging from 7 Å to 8.5 Å, with window sizes of 4 Å, diffusion, more so than reaction kinetics, could be the limiting step in the CWA destruction.

A formula unit (f.u.) of UiO-66 has one SBU containing six Zr atoms, four $\mu_{3}$-O atoms, and four $\mu_{3}$-OH groups, coordinated by 12 benzene dicarboxylate linkers. Each linker is shared between two SBUs. The $\mu_{3}$-OH groups are potential hydrogen bonding sites for polar guest molecules, including many CWAs. Dehydroxylated UiO-66, created by heating the material to high temperatures, has been explored elsewhere but is not considered here, as it is not representative of practical conditions for our desired applications [20].

Diffusion of guest molecules within UiO-66 has been studied both experimentally and with computer simulations [21,22,23,24,25,26,27]. In this work, we present self, corrected, and transport diffusion calculations of isopropyl alcohol (IPA) in pristine UiO-66. Self-diffusivities are computed from the Einstein relation [28],

(1) $$D_{S}=\lim_{t\to \infty }\frac{1}{2tdN}\langle \sum {\left|r_{i}\left(t\right)-r_{i}\left(0\right)\right|}^{2}\rangle ,$$

while the corrected diffusivities are calculated from the collective center of mass motion of all guest molecules,

(2) $$D_{c}=\lim_{t\to \infty }\frac{1}{2tdN}\langle {\left|\sum_{i=0}^{N}r_{i}\left(t\right)-r_{i}\left(0\right)\right|}^{2}\rangle ,$$

where *t* is time, *d* is dimensionality, and *N* is the number of atoms. The angled brackets represent an ensemble average. Transport diffusivity is calculated by applying the thermodynamic correction factor to corrected diffusivity, $D_{c}$,

(3) $$D_{t}=D_{c}{\left(\frac{\partial \ln f}{\partial \ln c}\right)}_{T},$$

where *f* is the fugacity of the bulk fluid in equilibrium with the adsorbed fluid at concentration *c* and temperature *T*. The thermodynamic correction factor can be calculated from the equilibrium adsorption isotherm of the system. Note that $D_{S}$, $D_{c}$ and $D_{t}$ are equal in the limit of infinite dilution.

Previously, we studied the diffusion of acetone in pristine UiO-66 [25]. Acetone functions as a valuable benchmark molecule due to its small size, which facilitates facile diffusion in pristine UiO-66 in contrast to most CWAs. Acetone is a hydrogen bond acceptor and can therefore form hydrogen bonds with $\mu_{3}$-OH groups within UiO-66 but does not exhibit hydrogen bonding with itself. In this work, we use IPA as the guest molecule; since IPA is both a hydrogen bond acceptor and donor, we can study the impact of MOF-IPA hydrogen bonding as well as that of IPA–IPA hydrogen bonding on diffusion. Recent work from Wang et al. also examined the transport properties of IPA in UiO-66 [23]. However, they used a MOF potential that did not allow for $\mu_{3}$-OH–IPA hydrogen bond formation.

Our previous work with acetone and UiO-66 has also shown the importance of including framework flexibility for accurate modeling of diffusion in UiO-66. The use of rigid force fields is popular for MOFs with large windows due to the added computational efficiency with limited sacrifice in accuracy [29]. For UiO-66, the difference in diffusivity between rigid and flexible models was shown to be essential for the accurate modeling of the diffusion of acetone [25].

Given the size of CWAs and their simulants, the study of adsorption and diffusion in defective UiO-66 is critical. The importance of defects in several applications of MOFs has been demonstrated [30,31]. This includes the adsorption of water in UiO-66, where defects were shown to promote adsorption, also influencing the favored binding sites of water with the MOF [32]. The intentional removal of linkers and/or metal clusters is anticipated to generate defects that would enhance the mobility and chemical reactivity of CWA with UiO-66. Furthermore, to obtain precise comparisons with experimental outcomes, the presence of defects is crucial, as defects are present in real materials.

Nevertheless, a more comprehensive understanding of the possible interactions between guests and pristine UiO-66 is valuable for understanding experiments on nearly pristine UiO-66 and for guiding experimental work on tailoring defects in MOFs. Specifically, knowledge of the pristine MOF enables the making of well-informed decisions regarding the placement of linker defects and the use of capping groups that best promote the desired application of the material.

In this study, we incorporated hydrogen bonding interactions between adsorbent and adsorbate (MOF-IPA) and between adsorbate and adsorbate (IPA-IPA) to understand their impact on the transport of IPA molecules through pristine UiO-66. We also checked the dependence of self, corrected, and transport diffusivities on the concentration of IPA on the pores of the MOF. These calculations were performed with a flexible framework potential, which has been shown to be critical for accurate diffusion calculations [25]. We compared our $D_{S}$ values calculated directly from molecular dynamics (MD) using the mean squared displacement method with values estimated from dynamically corrected transition state theory (dcTST). Our comparison revealed the expected accuracy of dcTST calculations for $D_{S}$ values of CWA molecules in UiO-66, which cannot be computed directly from MD simulations. We further investigated the importance of including hydrogen bonding interactions between the guest molecules and the framework by comparing the results when the guest-framework hydrogen bonding was turned off. We also measured the fraction of IPA molecules hydrogen-bonded with the framework. Our analysis provides key insights into the diffusion mechanism.

## 2. Materials and Methods

We used the Large-Scale Atomic/Molecular Massively Parallel Simulator (LAMMPS) to carry out MD calculations [33]. Simulations followed a method previously used with this same MOF and different adsorbates [25]. Periodic boundaries and a 12.5 Å cutoff were applied, with a timestep of 0.5 fs. Guest molecules were first randomly inserted into a simulation box containing the UiO-66 supercell. Then, the system was equilibrated for 50 ps in the canonical NVT ensemble with the Nosé–Hoover thermostat [34,35]. Finally, IPA mean squared displacements (MSDs) and trajectories were collected every 100 timesteps over a 25 ns production run in the microcanonical NVE ensemble.

A cubic UiO-66 supercell was constructed from a 2 × 2 × 2 replication of the unit cell and contained 32 octahedral and 64 tetrahedral cages. Half of the tetrahedral cages contained only $\mu_{3}$-OH groups and the other half only $\mu_{3}$-O groups. To evaluate the impact of the size of the supercell on the calculated diffusivities, we carried out test calculations with a 3 × 3 × 3 supercell.

We previously showed that the Rogge et al. [36] force field for UiO-66 does not account for hydrogen bonding between the $\mu_{3}$-OH groups on the MOF and guest molecules [25]. We therefore developed a modified potential that allows for guest–host hydrogen bonding; this modified potential is herein referred to as the Rogge/TraPPE potential and is used for most of the calculations in this work. We used the original Rogge et al. potential to compute the diffusion coefficient at zero loading to explore the impact of IPA–UiO-66 hydrogen bonding interactions on diffusion. We modeled IPA using both united-atom and all-atom models. We employed the TraPPE-UA [37] and OPLS-AA [38] potentials for united-atom and all-atom models, respectively.

We calculated self, corrected, and transport diffusivities. Self-diffusivities were estimated with the Einstein relation, Equation (1), by collecting MSD values computed internally from LAMMPS. Corrected diffusivities were calculated directly from the trajectories of guest molecules, with a method described in depth previously [39]. For each trajectory, 250 evenly spaced multiple-time origins were used to process the data, and results were batch-averaged over 20–30 total independent runs, batched into groups of 5. To test the convergence of our calculations we provide plots of MSD divided by time verses time for self and corrected diffusivities are given in Figures S1 and S2, respectively. We calculated transport diffusion coefficients by multiplying corrected diffusivities by the appropriate thermodynamic correction factors. The thermodynamic correction factors were calculated from IPA adsorption isotherms, with framework flexibility [40] being accounted for. The IPA adsorption isotherms were fitted to the dual-site Langmuir isotherm model. We calculated derivatives from the fitted isotherm to obtain the thermodynamic correction factor as a function of loading. Details about adsorption isotherm calculations can be found in the Supplementary Materials.

Diffusivity calculations were performed for zero and finite loadings. For zero loading, 100 noninteracting guest molecules were inserted into the UiO-66 supercell to achieve good statistics. Zero-loading simulations were carried out at 325 K, 350 K, and 425 K. From these calculations, the activation energy of diffusion was estimated using the Arrhenius equation:

(4) $$D=D_{0}\exp\left(\frac{-E_{a}}{RT}\right)$$

Finite loading simulations were performed using 1, 2, 3, 5, and 7 molecules per primitive cell (corresponding to 32, 64, 96, 160, and 224 molecules per 2 × 2 × 2 supercell, respectively). These values were chosen to span a range of pressures on the adsorption isotherm (Figure S3) from very low pressure to a loading that was slightly beyond the saturation loading (the loading at the saturation pressure estimated to be 6.8 IPA/f.u., as shown in Figure S3). All finite loading calculations were performed at 325 K.

For comparison, we also estimated zero-loading self-diffusivities at 325 K using the dynamically corrected transition state theory (dcTST) [41,42,43]. dcTST utilizes the rare event-sampling technique, umbrella sampling, instead of the conventional MSD method to estimate diffusion coefficients. The advantage of dcTST is that it can be employed to estimate the diffusion of larger molecules that do not diffuse in on time scales accessible to traditional MD. Our aim was to evaluate the accuracy of dcTST by comparing it with results from the MSD approach. The dcTST self-diffusivity, $D_{A\to B}$, is calculated by computing a value of diffusion along each possible hopping (diffusion) pathway as follows:

(5) $$D_{A\overset{}{\to }B}=\frac{1}{2d}k_{A\overset{}{\to }B}\lambda^{2},$$

where λ is the distance between sites *A* and *B*, which is the total distance along the reaction coordinate (RC), *d* is the dimensionality of the diffusion process, and $k_{A\overset{}{\to }B}$ is the hopping rate. The hopping rate is defined as

(6) $$k_{A\overset{}{\to }B}=\kappa \frac{1}{\sqrt{2\beta \pi m}}\frac{e^{-\beta F^{*}}}{\int_{\mathrm{state}\;A}e^{-\beta F(r)}dr},$$

where *m* is the adsorbate mass, $\beta =\frac{1}{k_{B}T}$, $k_{B}$ is the Boltzmann constant, F(r) is the free energy profile of the adsorbate along the reaction coordinate, $F^{*}$ is the activation-free energy, and κ is the dynamical correction factor, which accounts for short-time recrossings of the transition state. It defines the probability of the molecule settling to site *B* starting from the transition state [41,42]. The UiO-66 supercell has three different cages. These three cages share four pathways along which we computed the free energy barriers. We defined the RC as the path between the $\mu_{3}$-OH tetrahedral and octahedral and between the $\mu_{3}$-O tetrahedral and octahedral cages. Each pathway has a distance of λ=9.1 Å. The other two pathways are just the reverse (octahedral to tetrahedral). The overall $D_{S}$ value is calculated by combining the diffusivities along each of the possible pathways as follows:

(7) $$D_{S}=4\times {\left(\frac{1}{D_{A^{*}\overset{}{\to }B}}+\frac{1}{D_{B\overset{}{\to }A^{*}}}+\frac{1}{D_{A\overset{}{\to }B}}+\frac{1}{D_{B\overset{}{\to }A}}\right)}^{-1},$$

where state *A* denotes tetrahedral cages having $\mu_{3}$-O groups, $A^{*}$ denotes tetrahedral cages having $\mu_{3}$-OH groups, and *B* denotes octahedral cages.

Umbrella sampling was performed along these pathways to evaluate the free-energy landscape along each RC. We applied a biasing harmonic potential with a spring constant of 100 kcal mol${}^{-1}$ Å${}^{-2}$ to restrict the IPA molecule along the RC. Using a LAMMPS implementation of the Collective Variables package [44], we sampled umbrellas spaced at every 0.1 Å along the RC. We used a timestep of 0.5 fs for the sampling, starting with 50 ps NVT equilibration followed by 1 ns NVE production runs. We obtained free energies by performing the weighted histogram analysis method (WHAM) [45], which combines the resulting umbrellas into a single free-energy profile along the sampled pathway. The analysis yielded 4 free-energy profiles, 2 for each RC: a forward and a reverse pathway. Further, we calculated the transition state theory rate k${}_{\mathrm{TST}}$ ($s^{-1}$) from the obtained free-energy profiles. We followed previously implemented procedures [7,28,42,46,47] to calculate the value of the dynamical correction factor, κ. The adsorbate was restricted and sampled at the transition state with a harmonic bias potential spring constant of $10^{4}$ kcal mol${}^{-1}$ Å${}^{-2}$ for a 25 ps NVT equilibration and a 500 ps production run using a 1 fs timestep. The sampling recorded 2000 trajectories, which we used to run short MD simulations for a 10 ps production run starting with randomly assigned velocities at 325 K. The adsorbate position at the end of the simulations indicated whether the molecule was in a tetrahedral cage or an octahedral cage, yielding the dynamical correction factor, κ. We repeated this procedure for each pathway to acquire the dynamical correction factors. These are reported in Table S1.

We also computed binding energies, radial distribution functions (RDFs), and IPA density heatmaps in order to gain insight into the interactions influencing the diffusion process. More details on the binding energy and density heatmap calculations are given in the Supplementary Materials.

## 3. Results

Self-diffusivities for the Rogge/TraPPE potential were computed as a function of supercell size in order to explore finite-size effects. Finite cell size effects on diffusion are reported in Table 1. Results showed that the unit cell (1 × 1 × 1) is too small to give accurate diffusion coefficients. In contrast, results from the 2 × 2 × 2 and 3 × 3 × 3 supercells are identical within the uncertainties of the calculations. This shows that the 2 × 2 × 2 supercell is sufficiently large to give accurate diffusivities.

**Figure 1:**
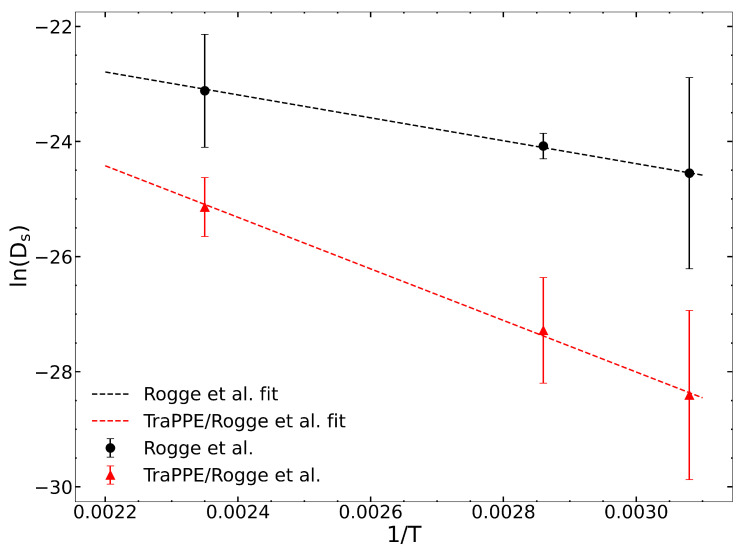
Arrhenius fit of the temperature-dependent zero-loading self-diffusion coefficients of IPA for the Rogge [36] et al. and Rogge/TraPPE UiO-66 potentials. The slope of the fitted line is equivalent to $E_{a}/R$, where *R* is the ideal gas constant.

We computed zero-loading diffusivities from the original Rogge et al. [36] and the Rogge/TraPPE UiO-66 potentials to evaluate the impact of guest–host hydrogen bonding. Self-diffusion coefficients are reported in Table 2 and are plotted in Figure 1. These data were fitted to the Arrhenius equation to calculate the diffusion activation energy ($E_{a}$) for the two potentials. The calculated values of the apparent activation energies are $E_{a}$ = 16.14 and 37.02 kJ/mol for the Rogge et al. and Rogge/TraPPE potentials, respectively. The difference of about 20 kJ/mol is due to the inclusion of hydrogen bonding between IPA and the $\mu_{3}$-OH group in the Rogge/TraPPE potential, but with this missing in the Rogge et al. potential.

**Figure 2:**
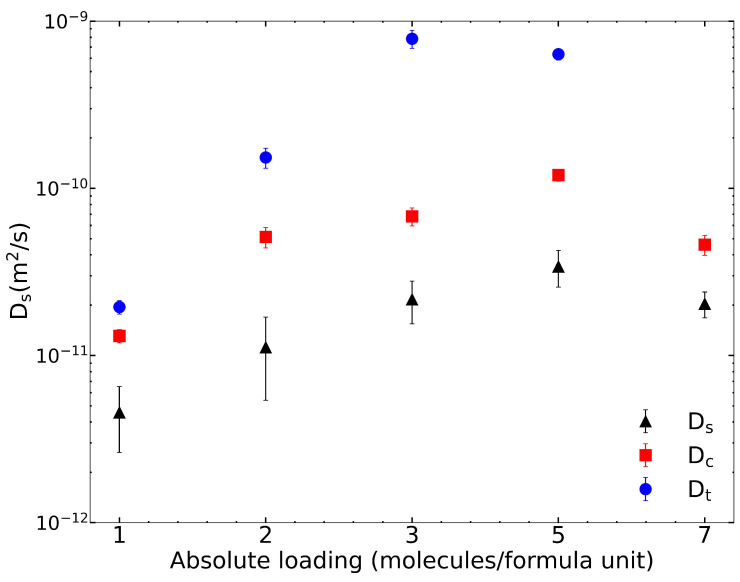
IPA diffusivity in pristine UiO-66 at finite loading. Self, corrected, and transport diffusion coefficients are reported. In comparison to that in Wang et al. [23], the self-diffusivity at seven loading has an order of magnitude difference.

We used the Rogge/TraPPE UiO-66 potential for all other calculations in this work. The finite loading diffusivities for IPA in pristine UiO-66 are plotted in Figure 2 and summarized in Table 3. As the loading per f.u. increases, $D_{S}$ and $D_{C}$ increase. However, at 7 IPA loading, both $D_{S}$ and $D_{C}$ values decrease. Corrected diffusivities are uniformly larger than are self-diffusivities, and transport diffusivities are larger than are corrected diffusivities. The calculation of the thermodynamic correction factor based on fits to the isotherms with dual-site Langmuir model (see Figure S4) is described in the Supplementary Materials and plotted in Figure S5.

Wang et al. [23] have also calculated the self-diffusivity of IPA in UiO-66, focusing on the effect of missing linker defects. They used the Rogge et al. potential [36], which does not effectively describe framework–IPA hydrogen bonding, as we have noted previously. The self-diffusivity calculated by Wang et al. for the pristine structure at 300 K is 3.4 $\times 10^{-10}$ m${}^{2}$/s. The adsorption isotherm reported by Wang et al. has a saturation concentration of 20 molecules per unit cell for the pristine structure, which is 5 IPA per f.u. loading. Our calculated self-diffusivity value at 5 molecules per f.u. loading is 3.41 $\times 10^{-11}$ m${}^{2}$/s, which is an order of magnitude smaller than is that reported by Wang et al. [23]. Note that the calculations of Wang et al. were performed at 300 K while ours were at 325 K, which means that their $D_{S}$ would be even larger at 325 K, augmenting the difference noted here. Hence, we conclude that IPA–$\mu_{3}$–OH hydrogen bonding decreases $D_{S}$ by at least an order of magnitude at saturation loading. This is consistent with our observations at zero loading in Table 2.

We compared $D_{S}$ computed from the TraPPE-UA and the OPLS-AA IPA potentials. Our results are given in Table 4. This was done to evaluate the validity of the coarse-grained approach of treating methyl groups as united atoms for this application. We performed calculations at zero loading and three loading using the OPLS-AA model for comparison. At zero loading, the two potentials produced statistically identical diffusion coefficients. However, at three loading, the OPLS-AA force field resulted in a diffusivity roughly four times smaller than the TraPPE potential, which is outside the statistical uncertainty. This indicates that accounting for IPA–IPA CH${}_{3}$ hydrogen atom interactions results in slower diffusion at finite loading. The same trend was noted previously for bulk $D_{S}$ of *n*-triacontane computed from the TraPPE-UA and OPLS-AA models [48]. Kondratyuk et al. reported that the experimental value of $D_{S}$ for *n*-triacontane lies in between the TraPPE-UA and OPLS-AA values, but closer to the OPLS-AA value [48]. Lacking a clear experimental comparison for IPA in UiO-66, it is difficult to definitively say which potential is more appropriate for calculating diffusivity, but we assume that the all-atom model should be more accurate. If this is the case, our simulations overpredict the true diffusivities. Despite these discrepancies, the qualitative trends in diffusion are expected to be the same regardless of model choice.

**Figure 3:**
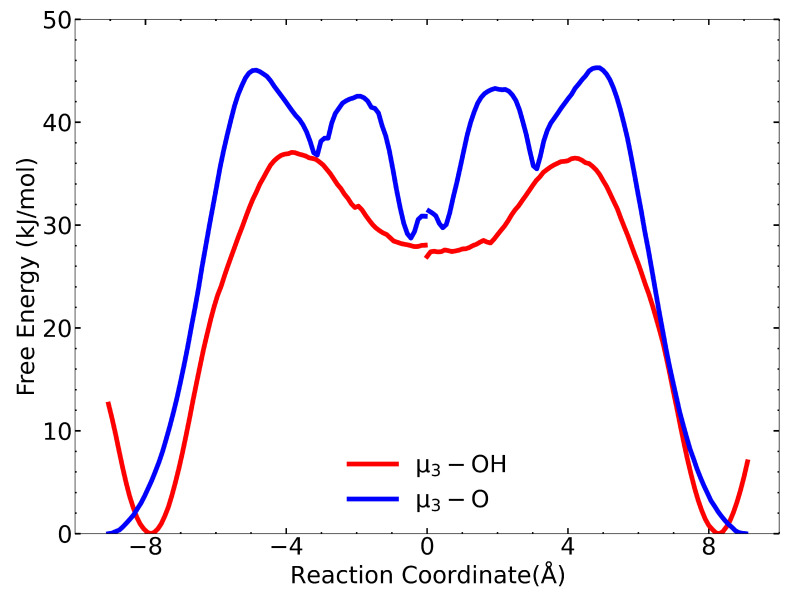
Free-energy barrier plot for tetrahedral-to-octahedral paths (left segment) and the converse paths (right segment). The tetrahedral $\mu_{3}$–OH path is shown in red, and the tetrahedral $\mu_{3}$–O path is shown in blue. The reaction coordinate is the distance between any point on the vector connecting the tetrahedral and octahedral cage centers (in Å). From left to right, the molecule travels from the tetrahedral cage to the octahedral cage (left segment) or in the opposite direction (right segment).

We used the dcTST method to estimate the diffusion coefficient at zero loading in order to compare them with the values we computed from the MSD approach. The free-energy profiles along all four RCs show that IPA has a lower free energy in the tetrahedral cages than in the octahedral cage, as seen in Figure 3. The energetic preference is due to the comparable sizes of IPA (kinetic diameter: five Å) and the diameter of the tetrahedral cage (seven Å). Hence, IPA has favorable interactions with many neighboring atoms in the framework for the tetrahedral pore. In contrast, the octahedral pore has a diameter of 8.3 Å, meaning that there are fewer nearest neighbor framework atoms with which the IPA can interact with compared with the tetrahedral pore. Figure 3 shows the free-energy profiles from the center of the tetrahedral cages to the center of the adjacent octahedral cage (forward pathway) and from the center of the octahedral cage to the center of the tetrahedral cages (reverse pathway) for both the $\mu_{3}$–OH and $\mu_{3}$–O tetrahedral cages. There is a discontinuity for the free-energy plots at RC = 0 Å because the forward and reverse paths come from two separate free-energy calculations. The forward and reverse paths should theoretically be perfect mirror images, and it is not necessary to calculate both since computing one gives the other. In practice, the differences in the forward and reverse path calculations give a measure of the statistical uncertainty (not accuracy) of the calculations. The maximum values in the free-energy profiles identify the transition states for IPA moving from one cage to the adjacent cage. In all cases, the transition state corresponds to the IPA molecule passing through the narrow triangular window, defined by three linker groups.

There are interesting differences between the free-energy plots in Figure 3 for the $\mu_{3}$–OH and $\mu_{3}$–O paths. The forward pathway for the $\mu_{3}$–OH path exhibits an initial decrease of about 12 kJ/mol in free energy occurring from −9.1 to −8.1 Å along the RC. After that, the free energy increases and reaches a maximum at about −4.5 Å. In contrast, no decrease in free energy is seen for the initial RC for the $\mu_{3}$–O path. We hypothesize that this difference is due to hydrogen bonding between the IPA O and the $\mu_{3}$–OH hydrogen; whereas, no hydrogen bonding can take place between IPA H and the $\mu_{3}$–O group because the O atom in the $\mu_{3}$–O moiety is sterically hindered by the surrounding Zr atoms [40]. This hypothesis is supported by our calculation of the free-energy profile from the $\mu_{3}$–OH tetrahedral to the octahedral cages using the original Rogge et al. potential, which is shown in Figure S6. As noted earlier, the Rogge et al. potential does not account for hydrogen bonding between IPA and the $\mu_{3}$–OH groups [25]. Note that in Figure S6, the Rogge et al. potential has only a very slight initial decrease along the RC, consistent with the $\mu_{3}$–O path, but very different from the $\mu_{3}$–OH path in Figure 3.

Another difference between the $\mu_{3}$–OH and $\mu_{3}$–O paths is that the barrier for the $\mu_{3}$–O tetrahedral to the octahedral cage is about 8 kJ/mol larger than the barrier for the $\mu_{3}$–OH path (barriers of 45 and 37 kJ/mol, respectively). In fact, we expect just the opposite, i.e., the barrier from the $\mu_{3}$–O tetrahedral cage to the octahedral cage should be lower based on the initial decrease in free energy as IPA moves from the center of the tetrahedral cage toward the SBU, where the $\mu_{3}$–OH group is located, as seen on both ends of the $\mu_{3}$–OH free-energy profiles in Figure 3. The decrease in free energy is due to the formation of a hydrogen bond between IPA and the $\mu_{3}$–OH moiety as we observed from visualization of the umbrella sampling trajectories at about −8 Å on the RC (not shown). In contrast, the barrier for the Rogge et al. potential for the $\mu_{3}$–OH tetrahedral to octahedral cage gives a barrier of about 26 kJ/mol, which is around 10 kJ/mol smaller than that with the Rogge/TraPPE potential and is consistent with the difference due to the hydrogen bonding free energy, inferred from the initial decrease seen in Figure 3. We have identified the likely reason for the higher barrier for the $\mu_{3}$–O pathway after careful analysis of the equilibrium geometry of the triangular windows defining the transition states for the $\mu_{3}$–OH and $\mu_{3}$–O cages. We examined the closest pairs of carbon atoms belonging to benzene moieties on neighboring BDC linkers, which define the size of the triangular window between the tetrahedral and octahedral pores (see Figure S9). We found that the windows on the $\mu_{3}$–O cages are slightly smaller than are the $\mu_{3}$–OH windows. The difference is only about 0.4 Å (see Table S4), but the smaller windows could translate into significantly larger barriers.

A third difference between these free-energy profiles is that the $\mu_{3}$–O tetrahedral to octahedral free energy plot shows double peaks, meaning that there are two transition states: the first occurring at a distance of about −5 Å along the RC and the second at about −2 Å. We suspect the double peaks are an artifact of the calculations, possibly involving instabilities in the IPA location since these are not present in similar calculations using the Rogge et al. potential (Figure S7).

The reverse pathway barriers, from the octahedral cage to each of the tetrahedral cages, are 10 kJ/mol and 16 kJ/mol for the tetrahedral $\mu_{3}$–OH and $\mu_{3}$–O cages, respectively. These calculations allow us to estimate the overall value of $D_{S}$ from Equation (7). Calculated data for all pathways are listed in Tables S2 and S3. Values for dynamical correction factors for all pathways are between 0.4 to 0.5. The dcTST method gives an estimate of $D_{S}=1.6\times 10^{-14}$ m${}^{2}$/s at 325 K. This value is over an order of magnitude smaller than the value computed from the MD simulations of $4.62\times 10^{-13}$ m${}^{2}$/s (Table 2). We also calculated $D_{S}$ at zero loading using the Rogge et al. potential. The free-energy pathways are shown in Figure S8. Our calculated value for $D_{S}$ is $7.88\times 10^{-15}$ m${}^{2}$/s, which is about 280 times smaller than is the value computed from the MSD of $2.18\times 10^{-11}$ m${}^{2}$/s reported in Table 2. This comparison gives an estimate of the accuracy of the dcTST method. The importance of this result is that we can assume $D_{S}$ values estimated from dcTST for larger molecules, such as CWAs and their simulants, diffusing in UiO-66 can be expected to be accurate to within about two orders of magnitude.

**Figure 4:**
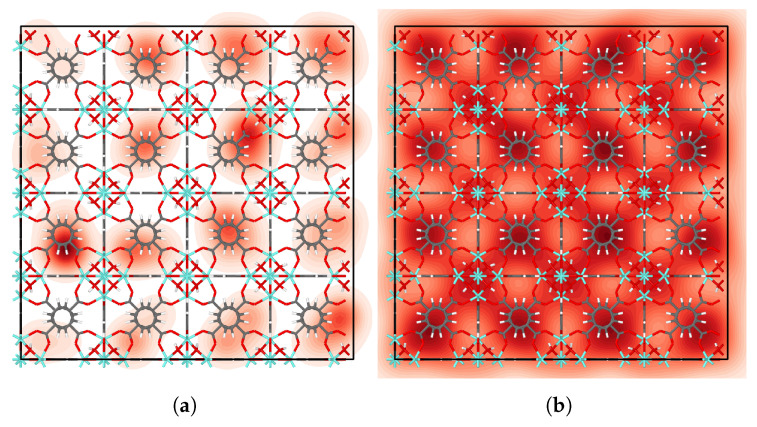
Distribution of IPA molecules in UiO-66 at (**a**) zero and (**b**) seven loading.

We examined the distribution of IPA molecules in UiO-66 by fitting a kernel density estimate to the center of mass positions of each molecule, averaged across all time steps. Further description of the approach is given in the Supplementary Materials. The resulting probability density plots (Figure 4, Figures S10 and S11) show that IPA molecules favor tetrahedral cages at low loading. For higher loading, IPA occupies octahedral cages as shown in Figure 4b. Moreover, the high-loading heatmap highlights the cage-to-cage transition pathways in very light red.

**Figure 5:**
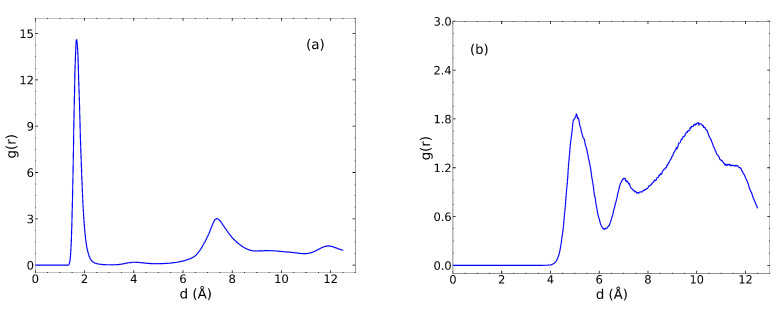
RDF plots between (**a**) the $\mu_{3}$–OH H atom and the IPA O atom and (**b**) between the $\mu_{3}$–O O atom and the IPA H atom.

We calculated the radial distribution function (RDF) for the IPA O atoms and UiO-66 $\mu_{3}$–OH H atoms, plotted in Figure 5a. The peak at 1.8 Å indicates a hydrogen bond between the IPA O and the H on the $\mu_{3}$–OH group. In contrast, the RDF for the IPA H and $\mu_{3}$–O O has a peak at about 5 Å (Figure 5b), indicating that IPA does not hydrogen bond with the $\mu_{3}$–O moiety. Qualitative evaluation of trajectories of the $\mu_{3}$–O region showed that Zr atoms surrounding the $\mu_{3}$-O oxygen sterically hinder the O atom from forming a hydrogen bond with the H atom of IPA. This is consistent with observations from a previous study on the adsorption of IPA in UiO-66 [40].

We estimated the ground state binding energies of IPA in each of the three unique cages of UiO-66 using the Rogge/TraPPE potential. This was shown in our previous work to agree relatively well with the binding energies calculated from DFT [25]. The results of IPA binding energies are presented in Table 5 and are compared to previous work with acetone as a reference [25]. With acetone, the $\mu_{3}$–OH tetrahedral cage is significantly favored due to the accessible hydrogen bonding sites, followed by the $\mu_{3}$–O cage and finally the octahedral cage. Generally, the tetrahedral cage is expected to be favorable for any relatively small guest molecule, as reported by Agrawal et al. [41]. We observed this same trend with IPA. In comparison with acetone, IPA binding is stronger in both the tetrahedral cages (by about 7 kJ/mol), but otherwise shows similar trends.

It is informative to compare and contrast the IPA binding energies with the free-energy differences computed from WHAM and reported in Figure 3. The binding energies are computed at zero Kelvin. The free energies computed from WHAM at 325 K include energetic and entropic effects; hence, the two quantities cannot be directly compared. The reference of energies for the $\mu_{3}$–OH and $\mu_{3}$–O paths in Figure 3 are different, so one cannot directly compare the relative free energies of IPA in the $\mu_{3}$–OH and $\mu_{3}$–O cages. However, differences in the free energies can be compared. The value of $\Delta A_{\mathrm{OT}}=A_{O}-A_{T}$ is the difference between the free energies of IPA in the octahedral and tetrahedral cages. We approximate this as the difference in the lowest free-energy value near the center of the octahedral pore (RC =0 in Figure 3) and the global minimum value (always zero by definition). For the $\mu_{3}$–OH pore, the difference $\Delta A_{\mathrm{OT}}$ is about 28 kJ/mol. For comparison, the difference in binding energies is $\Delta \Delta E_{\mathrm{bind}}=51.4$ kJ/mol (Table 5). We can approximate $\Delta A_{\mathrm{OT}}\approx \Delta \Delta E_{\mathrm{bind}}-T\Delta S_{\mathrm{OT}}$, with $\Delta S_{\mathrm{OT}}=S_{O}-S_{\mu_{3}-\mathrm{OH}}$, where $S_{O}$ and $S_{\mu_{3}-\mathrm{OH}}$ are the entropies of IPA in the octahedral and tetrahedral $\mu_{3}$–OH pores, respectively. Thus, the fact that $\Delta A_{\mathrm{OT}}<\Delta \Delta E_{\mathrm{bind}}$ means that $\Delta S_{\mathrm{OT}}>0$, which is what one would predict because of the larger volume for IPA to explore within the octahedral cage and the loss of entropy imposed on IPA by hydrogen bonding with the $\mu_{3}$–OH moieties. However, the difference of $\Delta A_{\mathrm{OT}}-\Delta \Delta E_{\mathrm{bind}}=-23.4$ kJ/mol is too large to be ascribed entirely to $\Delta S_{\mathrm{OT}}$, so it is likely that the WHAM calculations underestimate the true free-energy difference, $\Delta A_{\mathrm{OT}}$.

Considering now the $\mu_{3}$-O cage, we estimate $\Delta A_{\mathrm{OT}}=28$ to 30 kJ/mol, based on the uncertainty in the location of the minimum near RC=0 in Figure 3. The difference in the binding energies from Table 5 is −30.4−(−59.6)=29.9 kJ/mol, which is perhaps fortuitously close to our estimated value for $\Delta A_{\mathrm{OT}}$. The fact that the values of $\Delta A_{\mathrm{OT}}$ and $\Delta \Delta E_{\mathrm{bind}}$ are close indicates that $\Delta S_{\mathrm{OT}}=S_{O}-S_{\mu_{3}-O}\approx 0$. However, it is more likely that $\Delta S_{\mathrm{OT}}>0$ and that $\Delta A_{\mathrm{OT}}$ is somewhat overestimated for the $\mu_{3}$-O case. Despite the possible errors in the WHAM calculations, both the free energies and the binding energies indicate that the tetrahedral cages are preferred over the octahedral cages at low loading. This is confirmed by the density heatmap plots in Figure 4a and Figure S10.

We estimated the average fraction of IPA molecules engaged in IPA-$\mu_{3}$–OH and IPA–IPA hydrogen bonding as a function of loading (Figure S12). With increasing loading, the fraction of IPA-$\mu_{3}$–OH hydrogen bonds decreases and the fraction of IPA–IPA hydrogen bonding increases. We hypothesize that molecules involved in IPA-$\mu_{3}$–OH hydrogen bonds may diffuse more slowly than may others. To test this hypothesis, we computed MSDs of individual molecules, averaged over ten independent simulations. Histograms of the MSDs over 25 ns simulations are presented in Figures S13–S16 for IPA loadings of one, three, five, and seven. In each case, we observe a peak in the first histogram bin from 0 to 5 Å. We note that an MSD value <5 Å over the duration of the simulation indicates that the molecule is trapped in the cage; these molecules are therefore labeled as immobile. The fraction immobile IPA as a function of loading is reported in Table S5. The large fraction of immobile IPA at one loading (30%) is responsible for the relatively low value of $D_{S}$ compared with higher loading (Table 3). With increasing loading, the immobile IPA fraction decreases (Table S5). An et al. [24] noted that solid-state NMR data of IPA adsorbed in UiO-66 revealed a fraction of IPA molecules that do not change their binding environments over the time scale of the NMR experiment ($t_{1/2}=23$ μs). Their observations are consistent with our simulations showing immobile IPA although the time scale of our simulations is three orders of magnitude shorter than that of the NMR experiments. We note that the decrease in the fraction of immobile IPA does not correlate with the trends in $D_{S}$ for five and seven loading since these have essentially the same fraction of immobile IPA, but the value of $D_{S}$ is significantly lower for seven compared with five loading (Table 3). There are two factors leading to the decrease in $D_{S}$ at higher loading: (1) the increase in IPA–IPA hydrogen bonding (Figure S12) and (2) the onset of jamming at seven loading since the pores are completely filled.

## 4. Conclusions

In this work, we studied the diffusion of a polar molecule, IPA, in UiO-66. We calculated the self, corrected, and transport diffusivities as a function of the loading of IPA in UiO-66. We demonstrated that it is crucial to account for guest–host hydrogen bonding by comparing results for potentials that do or do not allow for IPA-$\mu_{3}$–OH hydrogen bonding. The values of $D_{S}$ obtained when accounting for hydrogen bonding are about an order of magnitude lower than those without hydrogen bonding for all values of loading, and the apparent activation energies obtained from the Arrhenius plots show that the inclusion of hydrogen bonding increases the apparent activation energy by about 20 kJ/mol. Self, corrected, and transport diffusivities initially increase with increasing loading but decrease once saturation coverage is reached. Closer analysis revealed that IPA molecules create hydrogen bonds with themselves, creating a blocking effect inside MOF cages at higher loading. Binding energies were calculated for all three cages in the framework, in which IPA in the tetrahedral $\mu_{3}$–OH cage showed the strongest binding. The heatmaps for IPA at zero loading showed preferential adsorption in tetrahedral cages, confirming the trend observed in binding energy calculations. We analyzed MSD values for individual molecules and found that a fraction of the molecules were immobile. We hypothesize that the immobile molecules are hydrogen-bonded to the $\mu_{3}$–OH groups in the tetrahedral cages. The self-diffusion coefficients for the Rogge/TraPPE and Rogge et al. potentials at zero loading calculated from the dcTST method are one to two orders of magnitude slower than are the values calculated from MSD, indicating that dcTST is likely accurate to within about two orders of magnitude. Our results on diffusion in pristine UiO-66 will inform the future examination of defects in the framework and their impact on the diffusion and catalytic degradation of CWAs.

## Figures and Tables

**Table 1:** Evaluation of the impact of finite size effects on 0-loading IPA self-diffusivity at 325 K.

Supercell Size	$D_{S}$ (m${}^{2}$/s)
1 × 1 × 1	1.14 (85) × $10^{-13}$
2 × 2 × 2	5.02 (63) × $10^{-13}$
3 × 3 × 3	5.40 (104) × $10^{-13}$

**Table 2:** Diffusion coefficients $D_{S}$ (m${}^{2}$/s) of IPA at zero loading using the Rogge et al. and Rogge/TraPPE UiO-66 potentials.

T (K)	Rogge et al. [36]	Rogge/TraPPE
325	2.18 (19) × $10^{-11}$	4.62 (23) × $10^{-13}$
350	3.49 (80) × $10^{-11}$	1.42 (40) × $10^{-12}$
425	9.11 (26) × $10^{-11}$	1.21 (20) × $10^{-11}$

**Table 3:** Diffusion coefficients of IPA at 325 K at finite loading computed from the Rogge/TraPPE potential.

Loading (N)	$D_{S}$ (m${}^{2}$/s)	$D_{C}$ (m${}^{2}$/s)	$D_{T}$ (m${}^{2}$/s)
1	4.57(194) × $10^{-12}$	1.31(12) × $10^{-11}$	1.95(18) $\times 10^{-11}$
2	1.12(58) × $10^{-11}$	5.1(72) × $10^{-11}$	1.53(21) × $10^{-10}$
3	2.17(62) × $10^{-11}$	6.8(84) × $10^{-11}$	7.85(97) × $10^{-10}$
5	3.41(84) × $10^{-11}$	1.20(94) × $10^{-10}$	6.35(49) × $10^{-10}$
7	2.04(36) × $10^{-11}$	4.6(63) × $10^{-11}$	NA

**Table 4:** Diffusion coefficients, $D_{S}$ (m${}^{2}$/s) computed from united-atom (TraPPE) and all-atom (OPLS-AA) potentials.

Loading	TraPPE	OPLS-AA
(molec./f.u.)		
0	4.62(23) × $10^{-13}$	3.53(84) × $10^{-13}$
3	2.17(62) × $10^{-11}$	5.63(115) × $10^{-12}$

**Table 5:** Binding energies of IPA (this work) and acetone [25] in UiO-66, computed from the Rogge/TraPPE potential. Relative differences in binding energies ($\Delta \Delta E_{\mathrm{bind}}$) are reported with the $\mu_{3}$–OH tetrahedral cage as the reference.

	IPA		Acetone [25]	
Cage	$\Delta E_{\mathrm{bind}}$	$\Delta \Delta E_{\mathrm{bind}}$	$\Delta E_{\mathrm{bind}}$	$\Delta \Delta E_{\mathrm{bind}}$
$\mu_{3}$–OH Tetrahedral	−81.7	0	−74.5	0
$\mu_{3}$–O Tetrahedral	−59.6	22.1	−52.6	22.8
Octahedral	−30.4	51.4	−34.5	40.9

## Data Availability

Details of the force fields used, plots of mean squared displacements, details of the thermodynamic correction factor, dynamically corrected transition state theory details, heatmap distributions, radial distribution functions, comparison of immobile and mobile IPA (PDF), zip folder containing data files, force fields, and dcTST method information information.

## Supplementary Materials

The following supporting information can be downloaded at https://www.mdpi.com/article/10.3390/nano13111793/s1, Figure S1: Mean squared displacement (MSD) divided by time (in Å${}^{2}$/ps) vs time (ps) (a) for each run, and (b) combining all independent runs for 5 loading IPA.; Figure S2: Center of mass mean squared displacement (CM-MSD) for corrected diffusivities divided by time (in Å${}^{2}$/ps) vs time (ps) (a) for each run, and (b) combining all independent runs for 5-loading IPA.; Figure S3: Adsorption isotherms of IPA in UiO-66 at 291 K computed from rigid framework (red up triangles), empty flexible framework (green diamonds) and flexible framework loaded with 7 IPA molecules/f.u. (black squares).; Figure S4: Dual-site Langmuir adsorption isotherm fits (dotted lines) to IPA isotherms computed from 7 loaded IPA flexible framework (blue circles) and from the empty flexible framework (red squares).; Figure S5: Thermodynamic correction factors computed from the adsorption isotherm using the 7 loaded IPA flexible framework (blue circles) and from the isotherm computed using the empty flexible framework (red squares). The maximum value for 7 loaded IPA framework corresponds to 3 molecules per f.u. and decreases with increasing after that. We believe the 7-loaded flexible isotherm better represents the true physics of the problem; Figure S6: Comparison of free energy profiles, starting from tetrahedral $\mu_{3}$-OH to an octahedral cage with and without the TraPPE modification. The Rogge/TraPPE potential resulted in higher barrier energy compared to the original Rogge et al. potential. The 11 kJ/mol difference between peaks can be ascribed to the hydrogen bond free energy; Figure S7: Comparison of free energy profiles starting from tetrahedral $\mu_{3}$-O to an octahedral cage with and without the TraPPE modification. In contrast to the $\mu_{3}$-OH cage, Rogge/TraPPE potential resulted in lower free energy barrier energy compared to the original Rogge et al. potential. There is a 3 kJ/mol difference between peaks; Figure S8: Comparison of free energy profiles from the $\mu_{3}$-OH to octahedral and $\mu_{3}$-O to octahedral pathways computed from the Rogge et al. potential; Figure S9: Schematic of the window connecting tetrahedral and octahedral cages. Elements C, H, O, and Zr are represented by grey, white, red, and cyan, respectively. Each window is formed by three linkers; 1,2,3. To measure the window aperture, we considered the distance between carbon atoms of adjacent linkers. The C1-C1 distance is shorter than the C2-C2 distance as shown in the schematic; Figure S10: Distribution of IPA molecules in UiO-66 at 1 Loading; Figure S11: Distribution of IPA molecules in UiO-66 at 3 Loading; Figure S12: Hydrogen bonding fractions as a function of loading for (a) IPA-$\mu_{3}$-OH and (b) IPA-IPA; Figure S13: Histogram of individual IPA molecule MSD at 1 loading. The ensemble average is given by the red line. Very fast-moving IPA (outliers above 600 Å${}^{2}$) are excluded from this figure; Figure S14: Histogram of individual IPA molecule MSD at 3 loading. The ensemble average is given by the red line. Very fast moving IPA (outliers above 600 Å${}^{2}$) are excluded from this figure; Figure S15: Histogram of individual IPA molecule MSD at 5 loading. The ensemble average is given by the red line. Very fast moving IPA (outliers above 600 Å${}^{2}$) are excluded from this figure; Figure S16: Histogram of individual IPA molecule MSD at 7 loading. The ensemble average is given by the red line. Very fast moving IPA (outliers above 600 Å${}^{2}$) are excluded from this figure. Table S1: Parameters for dual site Langmuir isotherms from the flexible snapshot method for empty and 7-loaded structures; Table S2: Dynamical correction factors, κ, hopping rates, $k_{A\to B}$, and $D_{S}$ values for all four paths. T denotes the tetrahedral cage and O denotes an octahedral cage; Table S3: Activation energies (in kJ/mol) for all paths defined for the dcTST calculations. Superscript f denotes the forward path, going from tetrahedral cage to octahedral cage, r denotes the reverse path, going from octahedral to tetrahedral cage; Table S4: Distances (in Å) between C-C atoms of adjacent BDC linkers making up the window between tetrahedral and octahedral cages in the equilibrium (ground state) structure of UiO-66. The three linkers making up the window are shown in Figure S9, which also gives the definition of the C1-C1 and C2-C2 carbon atoms. The column labeled “Path” refers to either the window from the $\mu_{3}$-OH to octrahedral or the $\mu_{3}$-O to octahedral cages. There are three pairs of linkers for which we have measured the C1-C1 and C2-C2 distances: Linker 1–Linker 3, Linker 1–Linker 2, and Linker 2–Linker 3. The distances are the same for each of the pairs, so we report the linker pair generically as Linker *i*–Linker *j*. Note that the $\mu_{3}$-OH cage has longer C-C distances than the tetrahedral $\mu_{3}$-O cage; Table S5: MSD analysis of IPA movement as a fraction of loading. The fraction of IPA with an MSD of less than 5 Å${}^{2}$ over 25 ns and the ensemble average MSD are reported. The former gives an indication of the fraction of IPA that remains in a single cage throughout the simulation. References [24,25,33,36,37,40,49,50,51,52] are cited in the supplementary materials.
