# Supplementary material for: Calculation of Self, Corrected, and Transport Diffusivities of Isopropyl Alcohol in UiO-66
Source: Nanomaterials (Basel). 2023 Jun 2;13(11):1793. doi: 10.3390/nano13111793 (PMC10254453; doi:10.3390/nano13111793)
Supplement: Supplementary file 1 [file nanomaterials-13-01793-s001.zip › nanomaterials-2421811 supplementary Info.pdf]

Supplementary Materials: Calculation of Self, Corrected, and Transport Diffusivities of Isopropyl Alcohol in UiO-66

Chinmay V. Mhatre [1,†], Jacob J. Wardzala [1,†,‡], Priyanka B. Shukla [1], Mayank Agrawal [2] and J. Karl Johnson [1,*]

1. UiO-66 Forcefield Specifications

In this work, UiO-66 is modeled by the classical forcefield first presented by Rogge et al. [1]. Covalent interactions are given by bond stretching, angle bending, and dihedral torsion terms. These covalent terms were developed using QuickFF. Non-bonded interactions are described by the MM3 potential with charge interactions governed by smooth Gaussian charge densities. 1-4 non-bonded LJ interactions and 1-2, 1-3, and 1-4 charge interactions were included. For adsorbate-framework cross interactions, LJ terms from UFF were used. As a result of previous work showing a failure in the Rogge potential to explain adsorbate-framework hydrogen bonding, LJ terms from the TraPPE [2] potential were used to describe μ_3-OH – adsorbate cross interactions. The parameterization of TraPPE was done in a manner that properly accounts for hydrogen bonding. Throughout the work, this modified potential is referred to as the Rogge/TraPPE potential.

2. Mean Squared Displacement

We present plots showing mean squared displacements (MSD) divided by time (MSD/t) for each individual run and the average of all 20 independent runs for computing D_S in Figure S1, and Figure S2. The slope of MSD/time vs time plot provides information about the extent of diffusion. The system is in the diffusive regime if the slope of MSD/time vs time is zero.

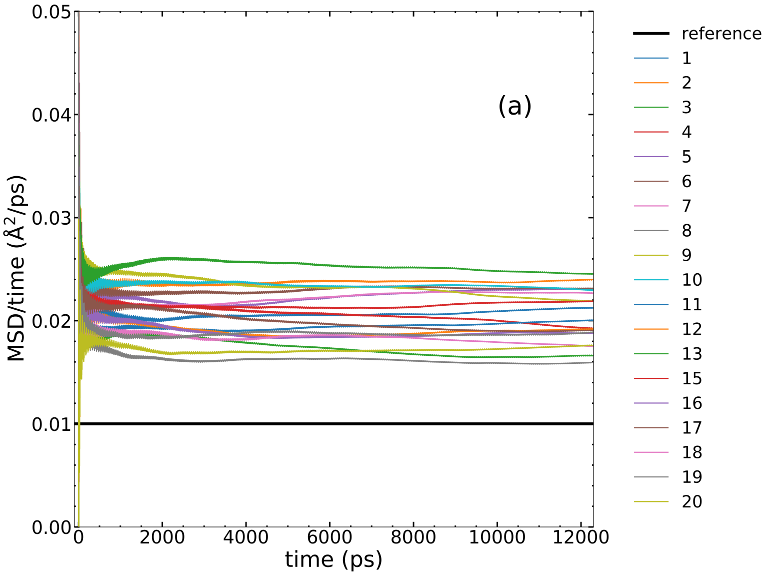

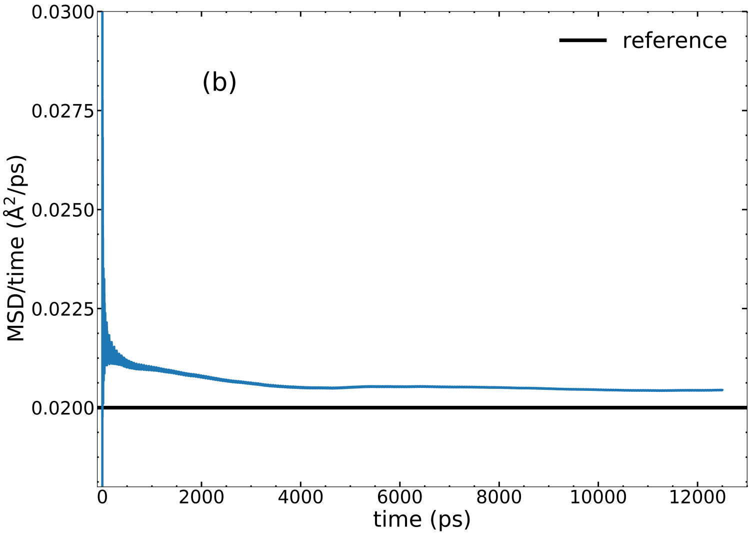

Figure S1. Mean squared displacement (MSD) divided by time (in $Å^2$/ps) vs time (ps) (a) for each run, and (b) combining all independent runs for 5 loading IPA.

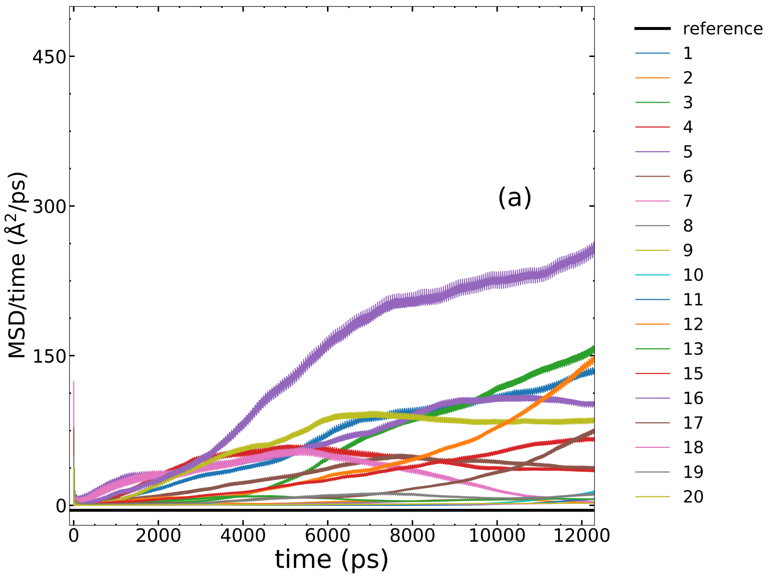

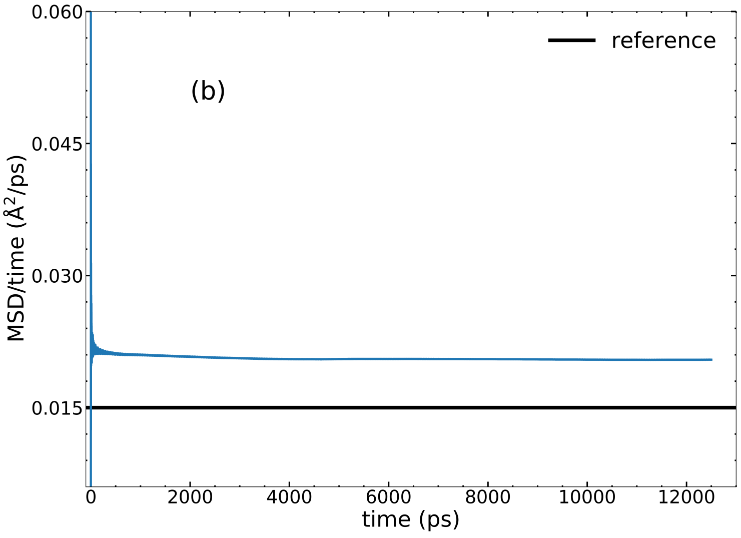

Figure S2. Center of mass mean squared displacement (CM-MSD) for corrected diffusivities divided by time (in Å^2/ps) vs time (ps) (a) for each run, and (b) combining all independent runs for 5-loading IPA.

3. Adsorption Isotherms

The adsorption isotherms of IPA in empty and adsorbate-loaded flexible UiO-66 were performed using a flexible snapshot method developed by Gee and Sholl [3], and which was modified by Shukla and Johnson to account for loading-dependence [4]. The flexible snapshot method performs GCMC simulations on several snapshots obtained from trajectories of NVT MD simulations, with each snapshot used as rigid structure in a GCMC simulation. The adsorption loading was computed as the average of the independent GCMC isotherms performed with MD-generated snapshots.

3.1. Flexible snapshot generation

A UiO-66 2×2×2 supercell containing 32 primitive cells was used to perform NVT MD simulations using the LAMMPS software package [5]. Each primitive cell contains two tetrahedral pores and one octahedral pore. Lattice parameters for the framework

are $a = b = c = 41.9568$Å $\alpha = \beta = \gamma = 90°$, and the cell volume = 73859.62 Å^3. NVT MD simulations were performed for empty and 7 IPA molecules per formula unit (f.u.) to generate snapshots to account for flexibility for adsorption calculations. The TraPPE-UA force field was used for IPA molecules. DDEC6 charges were computed using the Chargemol program for the framework atoms [6]. The MD simulations were performed with 1 fs timestep. The system was equilibrated for 100 ps using the Nosé-Hoover thermostat with a 0.1 ps decay period. Production runs were performed for 2 ns in the NVT ensemble, generating flexible snapshots every 200 ps, producing 10 uncorrelated NVT MD-generated snapshots. These snapshots were sufficient to compute adsorption isotherms in flexible frameworks, as shown previously [4].

3.2. GCMC simulations

We calculated adsorption isotherms of IPA in UiO-66 using the RASPA software suite [7]. We considered three different systems: (1) IPA in rigid UiO-66, (2) IPA in empty flexible UiO-66, and (3) IPA in flexible UiO-66 having structures generated with 7 IPA molecules/f.u. Then, we performed grand canonical Monte Carlo (GCMC) simulations on 10 NVT MD-generated snapshots of empty and IPA-loaded flexible frameworks (with IPA removed), while keeping framework atoms rigid during the simulation. These snapshots were averaged to obtain isotherms in flexible UiO-66. We simulated adsorption isotherms at 291 K to match previous experimental conditions [8], using 1×10^5 equilibration cycles and 70000 production cycles for each pressure in the isotherm. Each cycle contained N steps, where N is the number of molecules adsorbed during the cycle. We used the TraPPE-UA for IPA [2], the Universal Force field (UFF) [9] to calculate framework van der Waals interactions, and TraPPE Lennard-Jones parameters for OH moieties to compute adsorbate-adsorbent interactions and Lorentz-Berthelot combining rules for unlike pair interactions. We used DDEC6 charges for the framework atoms [6] and Ewald summations with default parameters to compute electrostatic potentials. We reported the helium void fraction for rigid, empty flexible, and flexible with 7 IPA molecules/f.u. in our previous work [4]. We calculated the ideal gas Rosenbluth weight for IPA to be 0.2411. We implemented swap, rotational, reinsertion, and translation moves in the ratio of 2:1:1:1. The calculated adsorption isotherms for IPA for the rigid, flexible empty, and flexible 7 loaded models are shown in Figure S3.

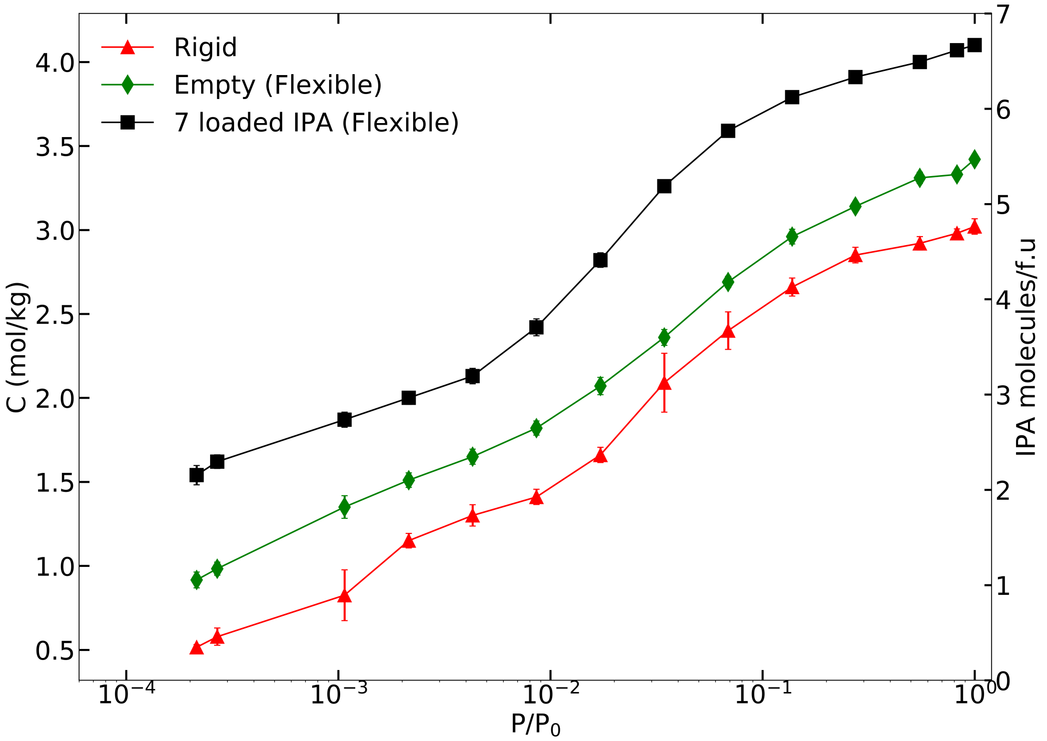

Figure S3. Adsorption isotherms of IPA in UiO-66 at 291 K computed from rigid framework (red up triangles), empty flexible framework (green diamonds) and flexible framework loaded with 7 IPA molecules/f.u. (black squares).

3.3. Thermodynamic Correction Factor

As described in the main text, we calculated the thermodynamic correction factor by taking the analytical derivative of the isotherm fit of c vs P. The dual-site Langmuir adsorption model, Equation (S1), was used to fit the adsorption data.

$$c = \frac{K_1 \times Q_1 \times \mathrm{P}}{1 + K_1 \times \mathrm{P}} + \frac{K_2 \times Q_2 \times \mathrm{P}}{1 + K_2 \times \mathrm{P}} \quad \text{(S1)}$$

Parameters for the fit are given in Table S1. Thermodynamic correction factors were evaluated for concentrations ranging from 1 molecule/f.u. to 5 molecules/f.u. with data from GCMC simulations. The adsorption isotherm of the empty flexible structure reaches saturation with 5.5 molecules/formula unit. Hence we could not report the transport diffusivity for 7 IPA loading.

Table S1. Parameters for dual-site Langmuir isotherms from the flexible snapshot method for empty and 7-loaded structures.

Structure	$K_1(Pa^{-1})$	$Q_1(cm^3/gm)$	$K_2(Pa^{-1})$	$Q_2(cm^3/gm)$
Empty	1.755	1.527	6.015×10^{-3}	1.922
7-loaded IPA	7.281	1.796	1.250×10^{-2}	2.320

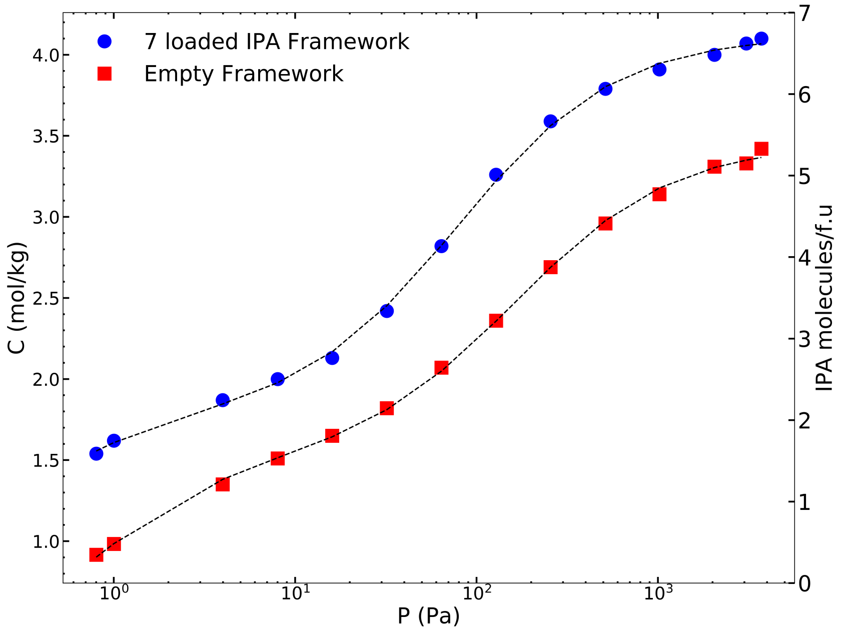

Figure S4. Dual-site Langmuir adsorption isotherm fits (dotted lines) to IPA isotherms computed from 7 loaded IPA flexible framework (blue circles) and from the empty flexible framework (red squares).

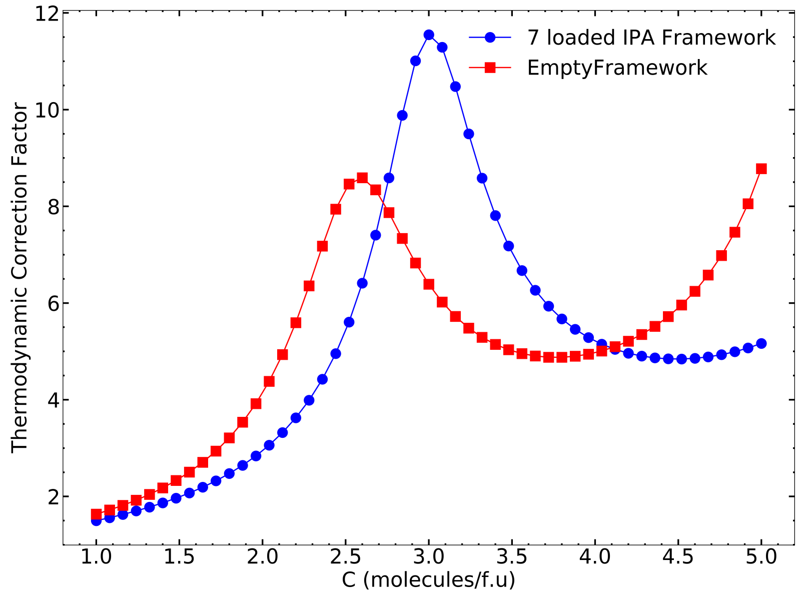

Figure S5. Thermodynamic correction factors computed from the adsorption isotherm using the 7 loaded IPA flexible framework (blue circles) and from the isotherm computed using the empty flexible framework (red squares). The maximum value for 7 loaded IPA framework corresponds to 3 molecules per f.u. and decreases with increasing after that. We believe the 7-loaded flexible isotherm better represents the true physics of the problem.

4. Dynamically Corrected Transition State Theory (dcTST)

The following section presents a comparison between the free energy barrier plot for Rogge et al. [1]. potential and Rogge/TraPPE corrected potential (Figure S6), and Table S2 with transition state rates, $k_{A\to B}$ and transmission coefficient, κ values for all four paths. Table S3 has listed activation energies of forward and reverse paths, used in the estimation of $k_{A\to B}$.

Table S2. Dynamical correction factors, κ, hopping rates, $k_{A\to B}$, and D_S values for all four paths. T denotes the tetrahedral cage and O denotes an octahedral cage.

	κ		$k_{A\to B}$ (s^{-1})		D_S (m^2/s)	
Rogge/TraPPE potential						
sampling path	μ_3-OH	μ_3-O	μ_3-OH	μ_3-O	μ_3-OH	μ_3-O
T → O	0.47	0.48	8.09×10^5	3.01×10^4	1.12×10^{-13}	4.15×10^{-15}
O → T	0.4	0.42	5.65×10^9	1.78×10^9	7.79×10^{-10}	2.45×10^{-10}
Rogge et al. potential						
T → O	0.5	0.51	2.11×10^7	1.43×10^4	2.9×10^{-12}	1.97×10^{-15}
O → T	0.41	0.46	4.04×10^9	1.15×10^9	5.6×10^{-10}	1.59×10^{-10}

Table S3. Activation energies (in kJ/mol) for all paths defined for the dcTST calculations. Superscript f denotes the forward path, going from tetrahedral cage to octahedral cage, r denotes the reverse path, going from octahedral to tetrahedral cage.

Path	E_A^f	E_A^r
Rogge/TraPPE		
μ_3-OH	37.02	9.56
μ_3-O	45.06	15.56
Rogge et al.		
μ_3-OH	26.45	10.34
μ_3-O	47.6	14.96

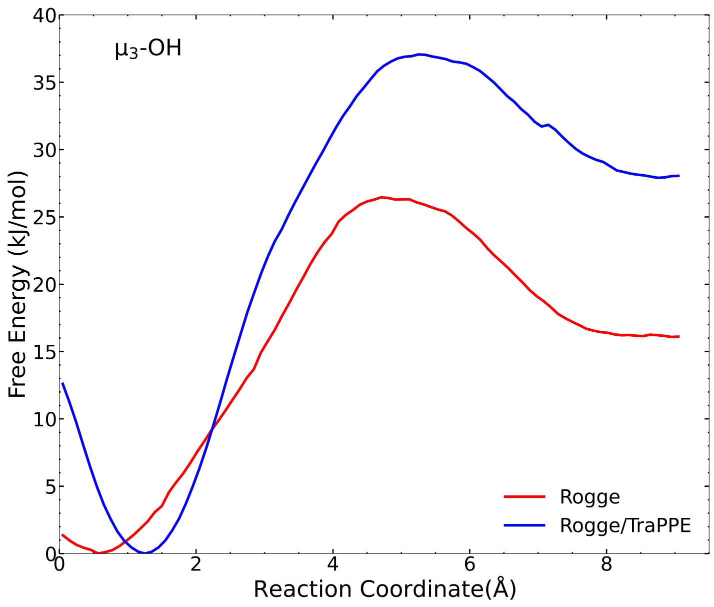

Figure S6. Comparison of free energy profiles, starting from tetrahedral μ_3-OH to an octahedral cage with and without the TraPPE modification. The Rogge/TraPPE potential resulted in higher barrier energy compared to the original Rogge et al. potential. The 11 kJ/mol difference between peaks can be ascribed to the hydrogen bond free energy.

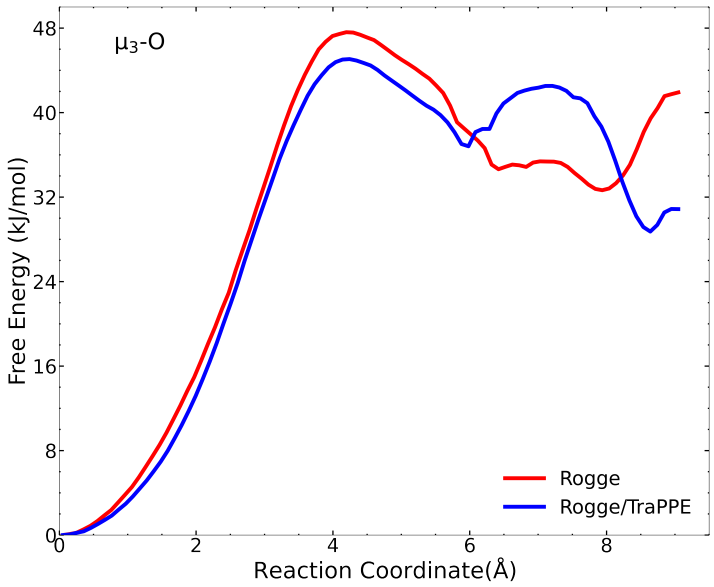

Figure S7. Comparison of free energy profiles starting from tetrahedral μ_3-O to an octahedral cage with and without the TraPPE modification. In contrast to the μ_3-OH cage, Rogge/TraPPE potential resulted in lower free energy barrier energy compared to the original Rogge et al. potential. There is a 3 kJ/mol difference between peaks.

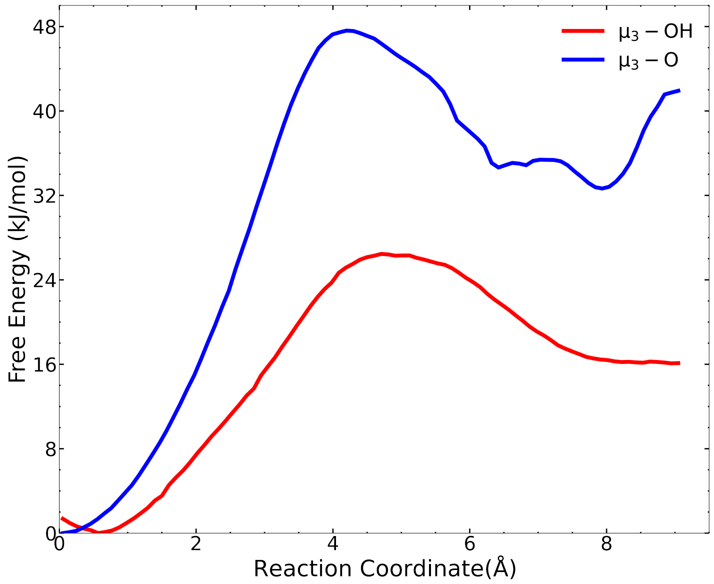

Figure S8. Comparison of free energy profiles from the μ_3-OH to octahedral and μ_3-O to octahedral pathways computed from the Rogge et al. [1] potential.

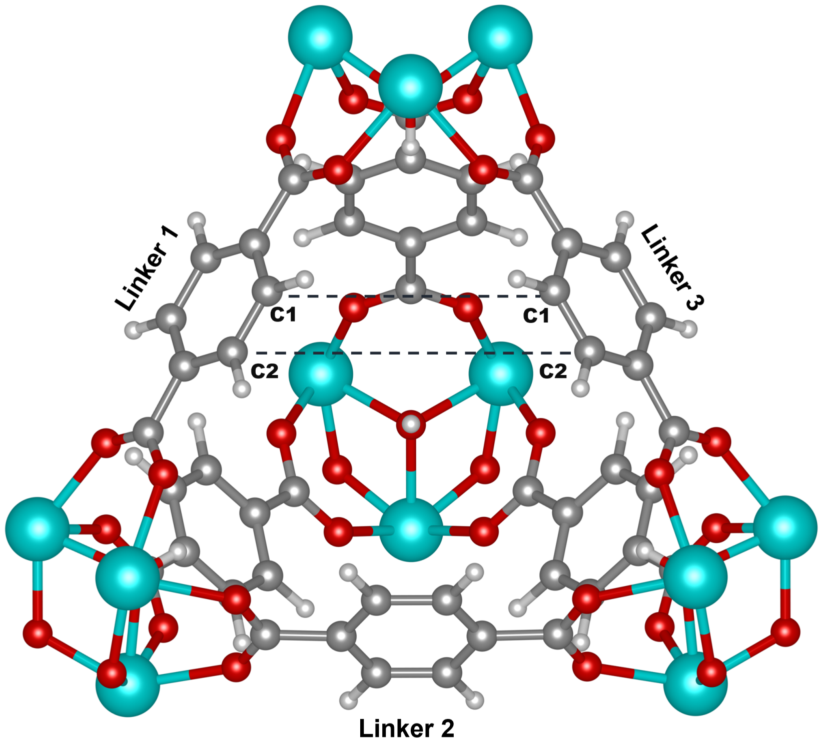

Figure S9. Schematic of the window connecting tetrahedral and octahedral cages. Elements C, H, O, and Zr are represented by grey, white, red, and cyan, respectively. Each window is formed by three linkers; 1,2,3. To measure the window aperture, we considered the distance between carbon atoms of adjacent linkers. The C1-C1 distance is shorter than the C2-C2 distance as shown in the schematic. Measured distances are reported in Table S4.

Table S4. Distances (in Å) between C-C atoms of adjacent BDC linkers making up the window between tetrahedral and octahedral cages in the equilibrium (ground state) structure of UiO-66. The three linkers making up the window are shown in Figure S9, which also gives the definition of the C1-C1 and C2-C2 carbon atoms. The column labeled "Path" refers to either the window from the μ_3-OH to octrahedral or the μ_3-O to octahedral cages. There are three pairs of linkers for which we have measured the C1-C1 and C2-C2 distances: Linker 1–Linker 3, Linker 1–Linker 2, and Linker 2–Linker 3. The distances are the same for each of the pairs, so we report the linker pair generically as Linker *i*–Linker *j*. Note that the μ_3-OH cage has longer C-C distances than the tetrahedral μ_3-O cage.

Path	Linker *i*–linker *j*
	C1-C1
μ_3-OH	5.7
μ_3-O	5.3
	C2-C2
μ_3-OH	7.1
μ_3-O	6.7

5. IPA Heatmaps

Distributions of IPA in UiO-66 across the range of examined loadings were visualized using kernel density estimation (KDE). The KDE serves as an estimate of the probability distribution function of the position of the center of mass of the IPA molecules. The produced distribution is shown plotted over a static image of the framework, which tracks IPA molecules inside the MOF.

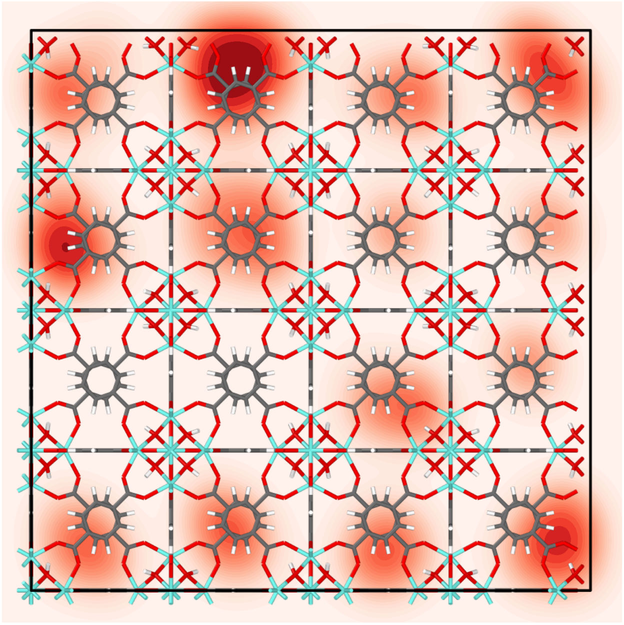

Figure S10. Distribution of IPA molecules in UiO-66 at 1 Loading.

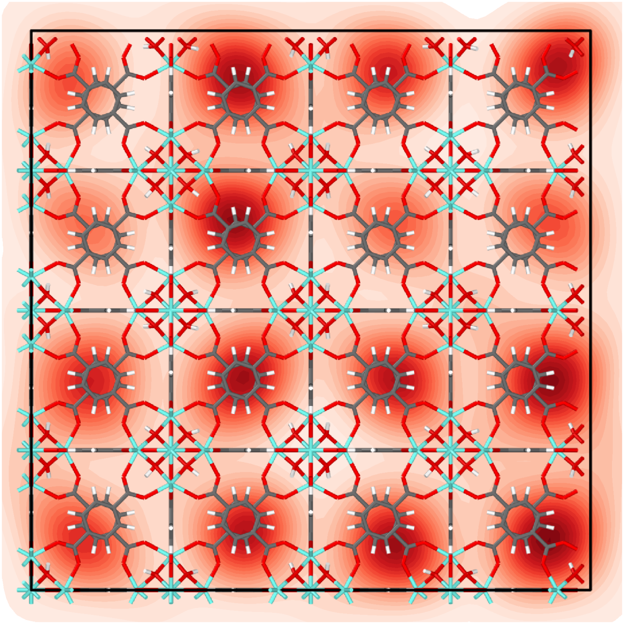

Figure S11. Distribution of IPA molecules in UiO-66 at 3 Loading.

6. Binding energy calculation

Binding energy calculations were performed using the Rogge/TraPPE corrected UiO-66 potential and IPA, by placing a single IPA molecule in the framework, following the methodology reported in previous work [10]. Only MOF, only IPA, and MOF+IPA systems were minimized in LAMMPS using a conjugate gradient minimizer. Many candidate configurations were sampled from the snapshots of an NVT simulation. The binding energies are defined as

$$\Delta E_{\text{bind}} = E_{\text{MOF+IPA}} - E_{\text{MOF}} - E_{\text{IPA}}, \tag{S2}$$

where each energy is relaxed to its local minimum. We used the lowest energy $E_{\text{MOF+IPA}}$ configuration as the ground state structure. The change in binding energy is defined as

$$\Delta\Delta E_{\text{bind}} = \Delta E_{\text{bind}}(\text{min}) - \Delta E_{\text{bind}}, \tag{S3}$$

where $\Delta E_{\text{bind}}(\text{min})$ is the binding energy with the lowest energy (global minimum).

7. Hydrogen Bonding

We examined the hydrogen bonding interactions to understand their effect on the diffusion process. These results are reported as fractions of the total IPA molecules engaged in two types of hydrogen bonds. The first is an interaction between the IPA oxygen and the μ_3-OH hydrogen. The second is a hydrogen bond between two IPA molecules. The results in Figure S12 show a decrease in the IPA-μ_3-OH hydrogen bonding fraction as loading increases, driven by the limited number of μ_3-OH groups with which IPA may form hydrogen bonds. The IPA-IPA hydrogen bonding fraction increases with increased IPA loading, as more IPA is available to interact with one another.

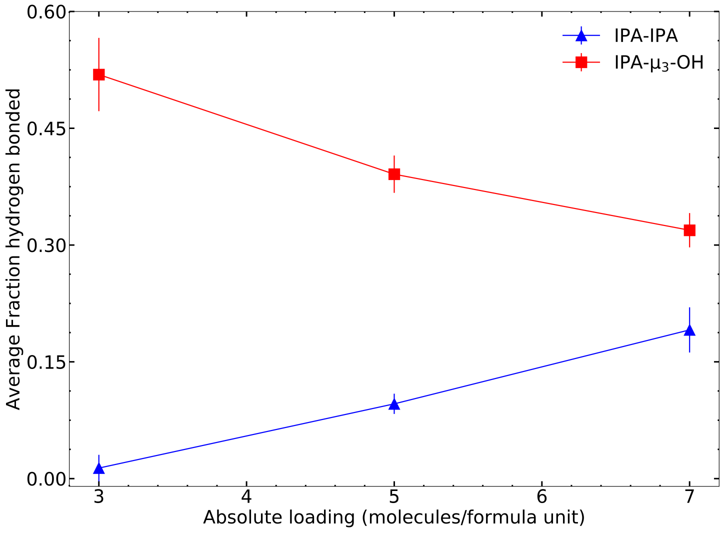

Figure S12. Hydrogen bonding fractions as a function of loading for (a) IPA-μ_3-OH and (b) IPA-IPA.

8. Mobile/Immobile Histograms

The individual IPA movement was tracked to complement the reported hydrogen bonding results. The MSD of these individual molecules was compared to the ensemble average and reported in Table S5. Specifically, the fraction of IPA having MSD < 5 Å^2 is tabulated. These IPA are essentially immobile throughout the simulations, with hydrogen bonds playing a crucial role in keeping them confined. The distributions of the IPA movements are displayed in the figures below. These distributions show a large number of immobile IPA, a smaller peak of IPA around the ensemble average, and then a smaller number of fast-moving IPA, which undergo repeated cage jumps throughout any single simulation.

Table S5. MSD analysis of IPA movement as a fraction of loading. The fraction of IPA with an MSD of less than 5 Å^2 over 25 ns and the ensemble average MSD are reported. The former gives an indication of the fraction of IPA that remains in a single cage throughout the simulation.

Loading	IPA with MSD less than 5 Å^2 (%)	Ensemble Average MSD (Å^2)
1	30.0	90.6
3	24.4	350.7
5	17.0	534.2
7	17.5	188.2

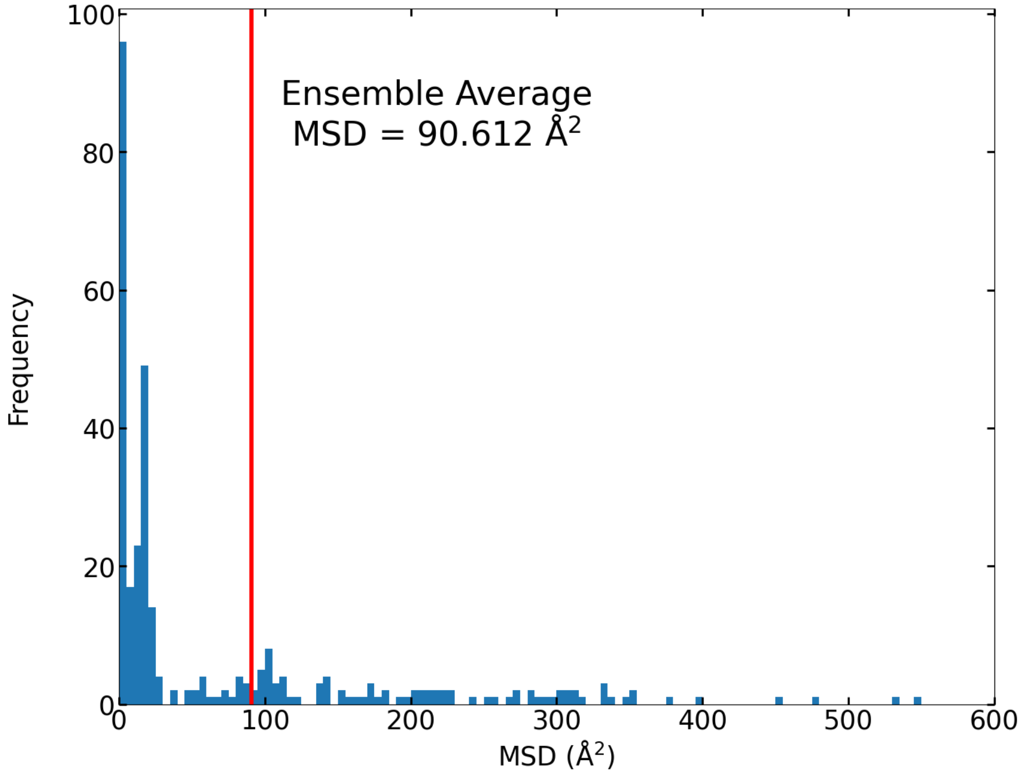

Figure S13. Histogram of individual IPA molecule MSD at 1 loading. The ensemble average is given by the red line. Very fast-moving IPA (outliers above 600 Å^2) are excluded from this figure.

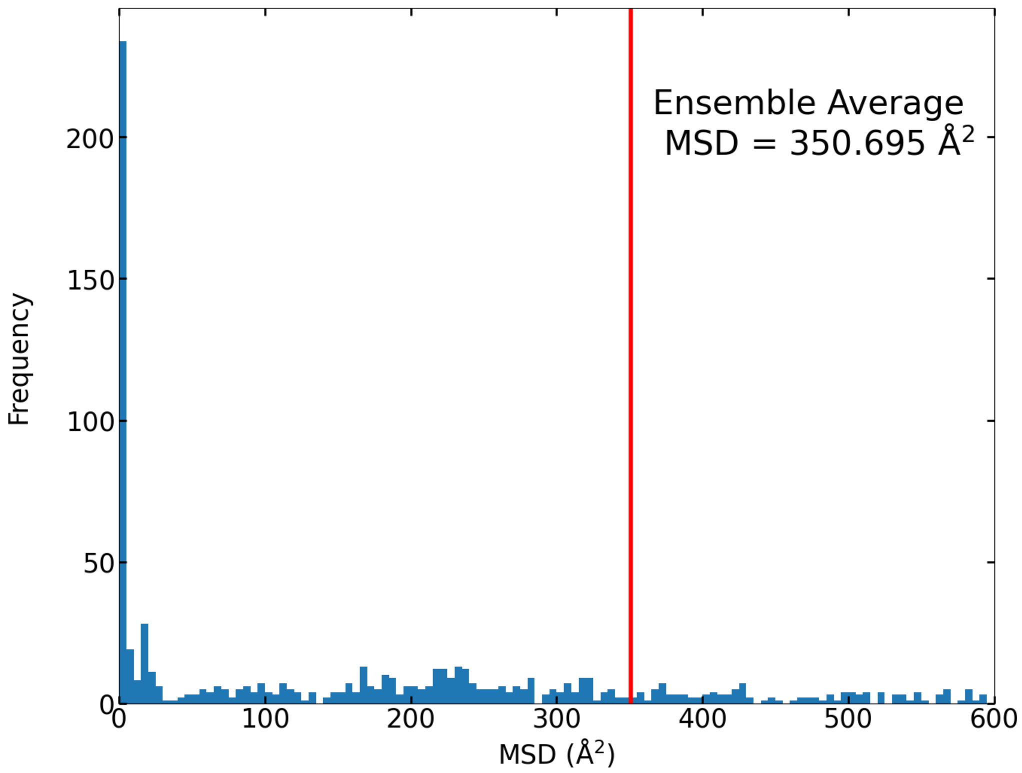

Figure S14. Histogram of individual IPA molecule MSD at 3 loading. The ensemble average is given by the red line. Very fast moving IPA (outliers above 600 Å^2) are excluded from this figure.

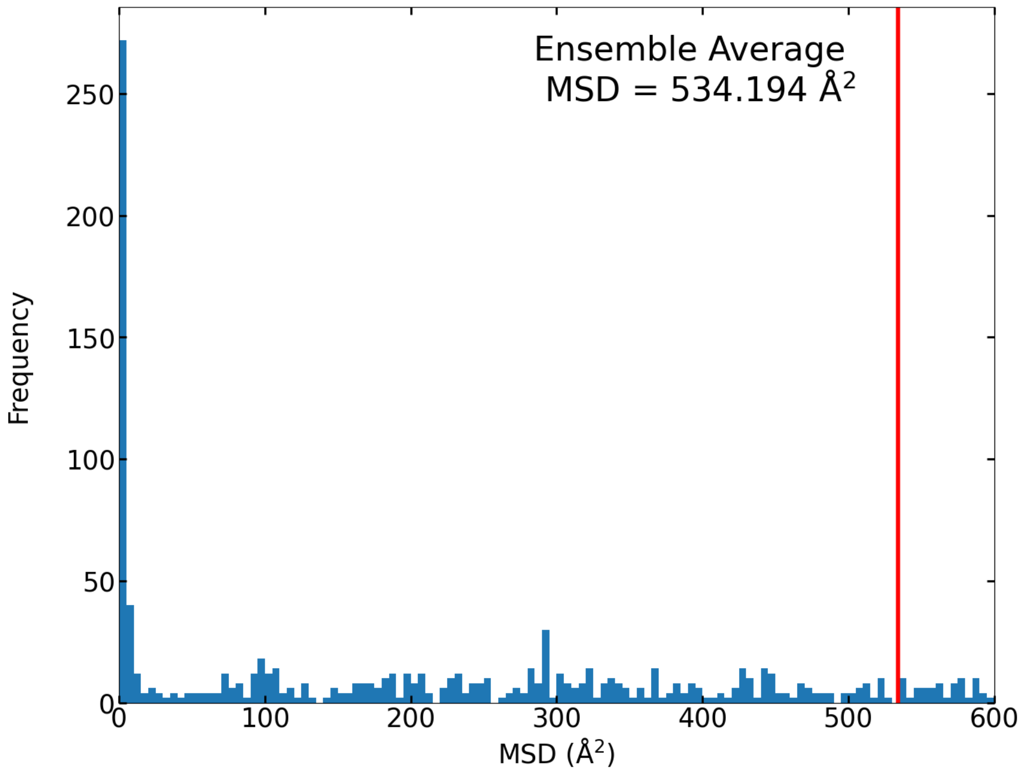

Figure S15. Histogram of individual IPA molecule MSD at 5 loading. The ensemble average is given by the red line. Very fast moving IPA (outliers above 600 Å^2) are excluded from this figure.

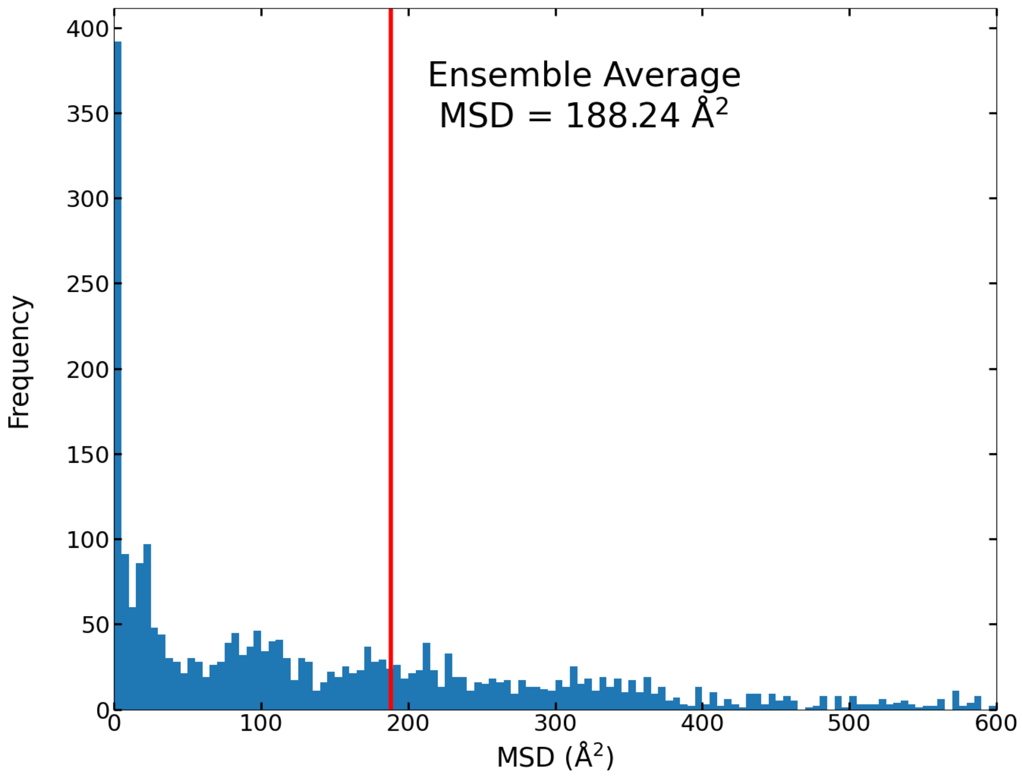

Figure S16. Histogram of individual IPA molecule MSD at 7 loading. The ensemble average is given by the red line. Very fast moving IPA (outliers above 600 Å^2) are excluded from this figure.

References

1. Rogge, S.M.J.; Wieme, J.; Vanduyfhuys, L.; Vandenbrande, S.; Maurin, G.; Verstraelen, T.; Waroquier, M.; Van Speybroeck, V. Thermodynamic Insight in the High-Pressure Behavior of UiO-66: Effect of Linker Defects and Linker Expansion. *Chemistry of Materials* **2016**, *28*, 5721–5732. https://doi.org/10.1021/acs.chemmater.6b01956.
2. Stubbs, J.M.; Potoff, J.J.; Siepmann, J.I. Transferable Potentials for Phase Equilibria. 6. United-Atom Description for Ethers, Glycols, Ketones, and Aldehydes. *The Journal of Physical Chemistry B* **2004**, *108*, 17596–17605. https://doi.org/10.1021/jp049459w.
3. Gee, J.A.; Chung, J.; Nair, S.; Sholl, D.S. Adsorption and Diffusion of Small Alcohols in Zeolitic Imidazolate Frameworks ZIF-8 and ZIF-90. *The Journal of Physical Chemistry C* **2013**, *117*, 3169–3176. https://doi.org/10.1021/jp312489w.
4. Shukla, P.B.; Johnson, J.K. Impact of Loading-Dependent Intrinsic Framework Flexibility on Adsorption in UiO-66. *The Journal of Physical Chemistry C* **2022**, *126*, 17699–17711. https://doi.org/10.1021/acs.jpcc.2c04629.
5. Plimpton, S. Fast Parallel Algorithms for Short-Range Molecular Dynamics. *Journal of Computational Physics* **1995**, *117*, 1–19. https://doi.org/10.1006/jcph.1995.1039.
6. Limas, N.G.; Manz, T.A. Introducing DDEC6 atomic population analysis: part 2. Computed results for a wide range of periodic and nonperiodic materials. *RSC Advances* **2016**, *6*, 45727–45747. https://doi.org/10.1039/c6ra05507a.
7. Dubbeldam, D.; Calero, S.; Ellis, D.E.; Snurr, R.Q. RASPA: molecular simulation software for adsorption and diffusion in flexible nanoporous materials. *Molecular Simulation* **2016**, *42*, 81–101. https://doi.org/10.1080/08927022.2015.1010082.
8. An, Y.; Kleinhammes, A.; Doyle, P.; Chen, E.Y.; Song, Y.; Morris, A.J.; Gibbons, B.; Cai, M.; Johnson, J.K.; Shukla, P.B.; et al. In Situ Nuclear Magnetic Resonance Investigation of Molecular Adsorption and Kinetics in Metal–Organic Framework UiO-66. *The Journal of Physical Chemistry Letters* **2021**, *12*, 892–899. https://doi.org/10.1021/acs.jpclett.0c03504.
9. Rappe, A.K.; Casewit, C.J.; Colwell, K.S.; Goddard, W.A.I.; Skiff, W.M. UFF, a full periodic table force field for molecular mechanics and molecular dynamics simulations. *Journal of the American Chemical Society* **1992**, *114*, 10024–10035, https://doi.org/10.1021/ja00051a040.
10. Wardzala, J.J.; Ruffley, J.P.; Goodenough, I.; Schmidt, A.M.; Shukla, P.B.; Wei, X.; Bagusetty, A.; De Souza, M.; Das, P.; Thompson, D.J.; et al. Modeling of Diffusion of Acetone in UiO-66. *The Journal of Physical Chemistry C* **2020**, *124*, 28469–28478. https://doi.org/10.1021/acs.jpcc.0c07040.
